# Novel Hantavirus Sequences in Shrew, Guinea

**DOI:** 10.3201/eid1303.061198

**Published:** 2007-03

**Authors:** Boris Klempa, Elisabeth Fichet-Calvet, Emilie Lecompte, Brita Auste, Vladimir Aniskin, Helga Meisel, Patrick Barrière, Lamine Koivogui, Jan ter Meulen, Detlev H. Krüger

**Affiliations:** * University Hospital Charité, Berlin, Germany; † Slovak Academy of Sciences, Bratislava, Slovak Republic; ‡ Museum National d'Histoire Naturelle, Paris, France; § Philipps University, Marburg, Germany; ¶ Severtsov Institute of Ecology and Evolution, Moscow, Russia; # Université de Rennes 1, Paimpont, France; ** Viral Hemorrhagic Fever Project, Conakry, Guinea; 1 Current affiliation: Leiden University Medical Center, Leiden, the Netherlands

**Keywords:** Hantavirus, shrew, Guinea, letter

**To the Editor:** Hantaviruses, family *Bunyaviridae*, have been known as causative agents of hemorrhagic fever with renal syndrome in Asia and Europe (*1*,*2*) and hantavirus cardiopulmonary syndrome in the Americas (*3*). Hantaviruses are spread by aerosolized rodent excreta and are strongly associated with their natural hosts, rodents of the family *Muridae*. Based on phylogenetic analyses, hantaviruses have been divided into 3 major groups that resemble 3 subfamilies of their natural hosts (Figure, panel A).

**Figure:**
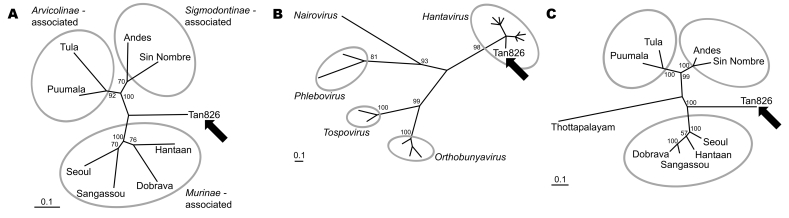
Maximum likelihood phylogenetic analysis of hantaviruses showing the phylogenetic placement of Tan826 (Tanganya virus, indicated by arrow) based on partial L segment nucleotide (A) and amino acid (B) sequences and partial S segment amino acid sequences (C); GenBank accession nos. EF050454 and EF050455, respectively. The values near the branches represent PUZZLE support values (*4*) calculated from 10,000 puzzling steps; only values ≥70% are shown. The scale bar indicates an evolutionary distance of 0.1 substitutions per position in the sequence. Gray ellipsoids indicate the 3 major hantavirus groups (panels A and C) or different genera of the *Bunyaviridae* family (panel B). We used 412 nt (137 aa) of the L segment (nucleotide position 2956–3367, amino acid position 974–1110) and 147 aa (nucleotide position 685–1111, amino acid position 217–364) of the putative nucleoprotein. Fragment positions were defined according to complete sequences of Hantaan virus strain 76-118 (GenBank accession nos. NC_005222 and NC_005218, respectively). The L segment fragment was amplified as described previously (*4*); for the S segment fragment, highly degenerated primers Cro2F (5′-AGYCCIGTIATGRGWGTIRTYGG-3′) and Cro2R (5′-AIGAYTGRTARAAIGAIGAYTTYTT-3′) were used. TREE-PUZZLE (www.tree-puzzle.de; [*4*]) was used to calculate the trees with HKY (panel A) and JTT (panels B and C) evolutionary models. Missing parameters were reconstructed from the data set. The following sequences were obtained from GenBank: X55901, AJ410617, X56492, DQ268652, M63194, AJ005637, L37901, AF291704, X14383, U12396, AF484424, AF133128, D10066, X56464, D10759, U15018 for L segment and M14626, L41916, S47716, DQ268650, M32750, Z69991, L25784, AF291702, AY526097 for S segment analysis.

Recently, we found the first indigenous African hantavirus, Sangassou virus (SANGV), in an African wood mouse (*Hylomyscus simus*) collected in Guinea (*5*). Thottapalayam virus (TPMV), isolated from an Asian house shrew (*Suncus murinus*) in India (*6*), is the only known hantavirus to be hosted by a shrew instead of a rodent (*7*,*8*). We report the recovery of hantavirus RNA of a novel sequence from a shrew, collected in Guinea, West Africa.

During a study of rodentborne hemorrhagic fever viruses performed in Guinea in 2002–2004, 32 shrews of the genus *Crocidura* were collected and screened for hantavirus RNA by reverse transcription–PCR (*5*). An RNA sample designated Tan826 produced a PCR product of the expected size. The animal host was a male *Crocidura theresae* collected in the grassland savannah around the village Tanganya (10°00′02″N, 10°58′22″W) in January 2004. Species identification, following the taxonomic nomenclature (*9*), was performed on the basis of morpho-anatomical characteristics and was supported by molecular analyses.

Partial L segment sequence of 412 nt was determined by cloning and sequencing of the obtained PCR product. Nucleotide sequence comparisons between Tan826 and other representatives of the genus *Hantavirus* showed very low sequence identity values, ranging from 67.7% (Andes virus) to 72.3% (Puumala virus). Corresponding sequences of deduced viral RNA polymerase (137 aa) showed only slightly higher similarity values of 69.3% (Tula virus) to 76.6% (SANGV). In a maximum likelihood phylogenetic tree (Figure, panel A), Tan826 did not unambiguously cluster with any of the major groups (i.e., *Murinae*-, *Arvicolinae*-, *Sigmodontinae*-associated viruses) and showed equal relatedness to all 3 groups. This exceptional position of the Tan826 sequence within the tree is consistent with its detection in a shrew instead of a rodent host. Because the sequence is only distantly related to other hantaviruses, sequences from additional members of the *Bunyaviridae* family were analyzed. Despite use of a suboptimal dataset of very divergent and short sequences, the phylogenetic placement of Tan862 within the genus *Hantavirus* could be clearly demonstrated (Figure, panel B).

Furthermore, a partial S segment sequence (442 nt, 147 aa of the putative nucleoprotein) was determined to compare Tan826 directly with the shrew-associated TPMV (for which only an S segment sequence was available in GenBank). Rather unexpectedly, the Tan826 sequence showed the lowest similarity to TPMV: 47.5% on nt level and 39.4% on aa level. The identity values to other *Hantavirus* members were also extremely low, 52.2% (Sin Nombre virus) to 62.1% (SANGV) on nt level and 50.6% (Andes virus) to 56.7% (Hantaan, Dobrava virus) on aa level. Corresponding aa sequences were then used for phylogenetic analysis to reduce problems derived from higher sequence diversities. In the resulting evolutionary tree, Tan826 and TPMV did not join any of the 3 major groups but also did not cluster together (Figure, panel C).

Our attempts to obtain more sequence data were hampered by the unique nature of the Tan826 virus sequence, which makes it difficult to design additional effective PCR primers, as well as by the limited amount of available biological material from the shrew. Nevertheless, the sequence and phylogenetic analyses of the 2 partial sequences strongly indicate that they represent a novel hantavirus. The amino acid sequences are highly divergent (≈25%–50%) from those of other hantaviruses and in phylogenetic trees; the Tan826 virus sequence appeared approximately equally related to those of all other hantaviruses. We propose to name the putative new species Tanganya virus (TGNV), after the locality where it was detected.

Detecting the virus in 1 of 32 *Crocidura* shrews, 15 of them *C. theresae*, is not sufficient to define *C. theresae* as a reservoir animal of this novel virus. However, the unique position of TGNV in evolutionary trees supports the idea that a shrew instead of a rodent is the natural host of TGNV. Therefore, it is rather surprising that TGNV did not form a monophyletic group with TPMV. Before this observation becomes either a challenge or support for the hantavirus–host coevolution concept, more extensive sequence data (for comprehensive phylogenetic analysis) and epizootiologic studies (to confirm the natural hosts of both viruses) are necessary.

TGNV represents, after the recently described SANGV (*5*), a second hantavirus from Africa. Its low sequence similarity to other hantaviruses should make this virus serologically distinct from other hantaviruses, as shown for TPMV (*10*). Therefore, human infections by TGNV might be missed when using antibody detection assays based on antigens from conventional hantaviruses.
